# Protocol for characterizing strigolactones released by plant roots

**DOI:** 10.1016/j.xpro.2022.101352

**Published:** 2022-05-18

**Authors:** Jian You Wang, Guan-Ting Erica Chen, Muhammad Jamil, Justine Braguy, Salim Sioud, Kit Xi Liew, Aparna Balakrishna, Salim Al-Babili

**Affiliations:** 1 The BioActives Lab, King Abdullah University of Science and Technology (KAUST), Thuwal, Saudi Arabia; 2 Center for Desert Agriculture, King Abdullah University of Science and Technology (KAUST), Thuwal, Saudi Arabia; 3 Plant Science Program, Biological and Environmental Science and Engineering Division, King Abdullah University of Science and Technology (KAUST), Thuwal, Saudi Arabia; 4 Analytical Chemistry Core Lab, King Abdullah University of Science and Technology (KAUST), Thuwal, Saudi Arabia

**Keywords:** Metabolism, Metabolomics, Model Organisms, Plant sciences, Mass Spectrometry

## Abstract

The plant hormone strigolactones (SLs) are secreted by plant roots to act as rhizospheric signals. Here, we present a protocol for characterizing plant-released SLs. We first outline all necessary steps required for collection, processing, and analysis of plant root exudates using the C_18_ column for SL extraction, followed by liquid chromatography-mass spectrometry (LC-MS) for SL quantification. We then describe image processing by SeedQuant, an open-source artificial-intelligence-based software, for measuring the biological activity of SLs in inducing root parasitic plant seed germination.

For complete details on the use and execution of this protocol, please refer to Wang et al. (2019) and Braguy et al. (2021).

## Highlights

• Protocol for identifying strigolactones (SLs) from exudates of plant roots
• Step-by-step instructions to analyze SLs by LC-MS and seed bioassay
• Can be applied to most of the plant species grown hydroponically or in sand

## Graphical abstract

**Figure:**
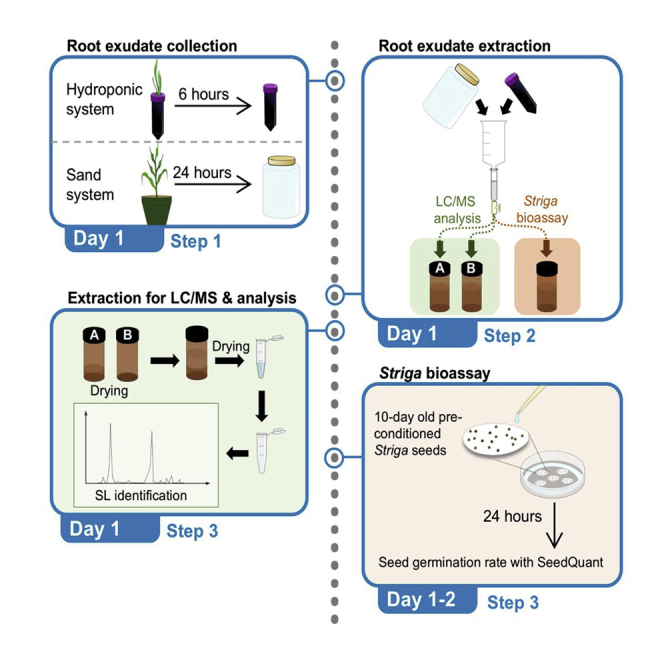

## Before you begin

Strigolactones (SLs) are evolutionarily conserved apocarotenoid metabolites (Walker et al., 2019; Wang et al., 2021) that originate from carotenoids cleavage (Alder et al., 2012). SLs regulate plant architecture (Gomez-Roldan et al., 2008; Umehara et al., 2008; Al-Babili and Bouwmeester, 2015), and, particularly in nutrient deficiency conditions, under which they are exuded by plant roots for establishing arbuscular mycorrhizal symbiosis (Al-Babili and Bouwmeester, 2015; Fiorilli et al., 2019). However, SLs are released at extremely low concentrations (∼picoMolar per liter of root exudate), even under phosphate starvation, and are relatively unstable, which makes their quantification difficult (Boutet-Mercey et al., 2018). The protocol below describes specifically and in a stepwise manner: the SL extraction from root exudates of rice (Wang et al., 2019, 2020) and pearl millet - when cultured in hydroponic and sand system - and their subsequent analysis using (1) the detection and quantification of SLs by LC-MS, as well as (2) the evaluation of their efficacy as seed germination stimulant of the root parasitic plant *Striga hermonthica,* using SeedQuant (Braguy et al., 2021). Our protocol is also suitable for many other plant species, such as Arabidopsis (Ablazov et al., 2020) and tomato; however, it may need some modifications with respect to the parameters of the MS analysis. In addition, testing the germination-inducing bioactivity may require changes in the seed preconditioning step (Matúšová et al., 2004) if other parasitic weeds are used.

### Preparation of growth mediums

**Timing: ∼2 h**

1. Preparation of ½ MS medium.
  a. Dissolve 2.21 g Murashige & Skoog (MS) Basal Medium (without vitamins) in 1 L Milli-Q water and adjust the pH to 5.8.
  b. Autoclave ½ MS medium for further use.
2. Preparation of half-strength modified Hoagland nutrient solution.
  a. To make +Pi solution (normal growth condition), add 1 mL of each stock solution, except 10 mL of iron EDTA (FeSO4), (Table 1) to 900 mL of Milli-Q water. Thereafter, add extra Milli-Q water to make the final volume 1 L.
    ***Note:*** Preparation of Hoagland nutrient solution stocks is listed in Table 2.
    ***Note:*** For the low Pi or -Pi conditions, the solution is made with 0.01 mL/L of 0.4 mM K_2_HPO_4_ or without 0.4 mM K_2_HPO_4_, respectively.

**Table 1:** Preparation of 1 L half-strength modified Hoagland nutrient solution

Reagent	Stock concentration	Final concentration	Amount
1. Ammonium nitrate (NH_4_NO_3_)	5.6 mM	5.6 μM	1 mL
2. Magnesium sulfate heptahydrate (MgSO_4_∗7H_2_O)	0.8 mM	0.8 μM	1 mL
3. Iron EDTA (FeSO_4_)	0.18 mM	1.8 μM	10 mL
4. Calcium chloride dihydrate (CaCl_2_.2H_2_O)	1.6 mM	1.6 μM	1 mL
5. Potassium nitrate (KNO_3_)	0.8 mM	0.8 μM	1 mL
6. Potassium phosphate dibasic trihydrate (K_2_HPO_4_.3H_2_O)	0.4 mM	0.4 μM	1 mL
7. Micronutrients∗∗	n/a	n/a	1 mL
ddH_2_O	n/a	n/a	984 mL
**Total**	**n/a**	**n/a**	**1,000 mL**

∗∗Micronutrient		
Reagent	Stock concentration	Final concentration
Boric Acid (H_3_BO_4_)	0.023 mM	11.4974 μM
Manganese chloride (MnCl_2_∗4H_2_O)	0.0045 mM	2.2484 μM
Copper Sulfate (CuSO_4_∗5H_2_O)	0.0003 mM	0.1481 μM
Zinc Chloride (ZnCl)	0.0015 mM	0.75 μM
Sodium molybdate (Na_2_MoO_4_∗2H)	0.0001 mM	0.0495 μM
  b. Adjust the pH to 5.8.
    ***Note:*** The pH of the half-strength modified Hoagland nutrient solution should be re-adjusted to 5.8 every time before using. The growth medium (½ MS medium and half-strength modified Hoagland nutrient solution) can be kept at room temperature (23°C–25°C) up to 1 month.
    **CRITICAL:** All the chemicals are dissolved in the following 7 stock solutions to avoid any precipitation. All the stock solutions should be stored at 4°C.
    **CRITICAL:** The stock solutions can be kept at 4°C up to 3 month.

**Table 2:** Stocks of half-strength modified Hoagland nutrient solution

Stock no.	Chemicals name	Concentration (mM)	Molecular weight (g/mol)	Mass (g/L)
1.	NH_4_NO_3_	5.6	80.04	224.1
2.	MgSO_4_.7H_2_O	0.8	246.48	98.6
	K_2_SO_4_	0.8	174.2	70.0
3.	FeSO_4_.7H_2_O	0.18	278.0	2.55
	Na_2_EDTA.2H_2_O	0.02	372.2	1.86
4.	CaCl_2_.2H_2_O	1.6	147.02	117.6
5.	KNO_3_	0.8	101.11	40.4
6.	K_2_HPO_4_.3H_2_O	0.4	228.2	50.0
7.	**Micronutrients**			
	H_3_BO_3_	0.023	61.84	0.711
	MnCl_2_.4H_2_O	0.0045	197.91	0.445
	CuSO_4_.5H_2_O	0.0003	249.68	0.037
	ZnCl_2_	0.0015	136.32	0.102
	Na_2_MoO_4_.2H_2_O	0.0001	241.95	0.012

1. NH_4_NO_3_; 2. K_2_HPO_4_.3H_2_O; 3. MgSO_4_+ K_2_SO_4_; 4. FeSO_4_.7H_2_O and Na_2_EDTA.2H_2_O; 5. CaCl_2_; 6. KNO_3_; 7. Micro nutrient.

### Plant growth

**Timing: Rice (*Oryza sativa* L.) as an example: 21 days for hydroponic; 42 days for sand**

3. Seed sterilization.
  a. Place the rice seeds (*Oryza sativa* L.) in a 50 mL falcon tube containing 50% commercial bleach (sodium hypochlorite) solution (Milli-Q water + commercial bleach; 1:1 v/v).
  b. Continuously shake the tube in a rotator for 10 min to sterilize the surface of the seeds.
    ***Note:*** An addition of 2 drops of Tween-20 to the 50% sodium hypochlorite solution can improve the seed sterilization step.
    ***Optional:*** For the sterilization of pearl millet (*Pennisetum glaucum*) seeds, do not add Tween-20 and sterilize for only 5 min.
  c. In a sanitized laminar flow cabinet, wash the seeds successively 5–6 times with sterilized Milli-Q water.
  d. For imbibition, keep the seeds in the falcon tube with 30 mL sterilized Milli-Q water overnight (∼12 h) in an incubator, in the dark at 30°C.
    ***Note:*** Pearl millet seeds can be directly placed in the Petri dish without imbibition (see step 4).

4. After imbibition, spread 10 rice seeds on a 100 mm × 15 mm Petri dish, containing two filter papers moistened with 5 mL of ½ MS medium.

***Note:*** Manipulate under sterile conditions in a laminar flow cabinet.

***Optional:*** For pearl millet, spread around 20 seeds per 100 mm × 15 mm Petri dish, containing two filter papers moistened with 5 mL of ½ MS medium.

5. Seal the Petri dishes with parafilm, and wrap them in aluminum foil to avoid exposure of light to the seeds.
6. Place the Petri dishes in a 30°C incubator for 2 days.
7. Remove the aluminum foil and the parafilm, and transfer the germinated seeds to a growth chamber with day/night temperature of 28/22°C and a 12 h photoperiod, 200 μmol photons m^−2^ s^−1^ for 5 days.

***Note:*** Check the seeds on the third day and add ½ MS medium if needed.

**Figure 1:**
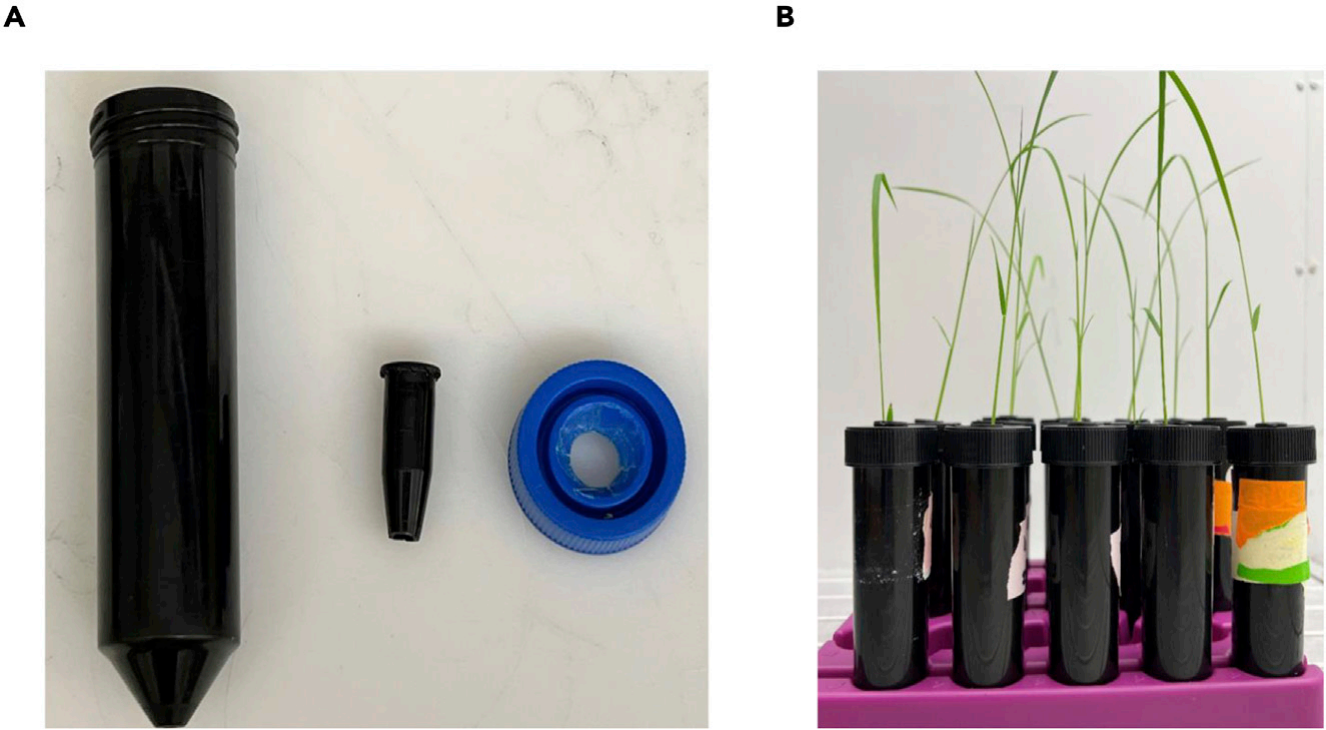
The setup for the hydroponic system of 50 mL falcon tube (A) Make one hole in the center of the cap of the 50 mL tube. Cut the bottom of a 1.5 mL lid-removed black Eppendorf tube and place it in the hole of the 50 mL tube’s cap. (B) A represented picture of rice seedlings grown under hydroponic system.
  c. Fill the 50 mL black tube with +Pi Hoagland nutrient solution.
  d. Transfer one-week-old rice seedlings (1–2) through the 1.5 mL bottomless Eppendorf tube in the center of each cap and fix the cap into 50 mL black tubes (Figure 1B).
  e. Keep the tubes with seedlings in a growth chamber for 1 week (+P) and refresh the nutrient solution two times per week.

8. Grow rice seedlings under hydroponic conditions.
  a. Make one hole in the center of the cap of 50 mL tube.
  b. Cut the bottom of a 1.5 mL Eppendorf tube, remove the lid if it has one, and place it in the hole of the 50 mL tube’s cap (Figure 1A).

***Optional:*** For pearl millet, transfer seven germinated seedlings to a 2 L pot filled with silver sand and apply 1 L +Pi half-strength modified Hoagland nutrient solution twice per week for 4 weeks.

9. Trigger the SL production and exudation by applying (–Pi) half-strength modified Hoagland nutrient solution for another 1 week (Wang et al., 2019).

**Figure 2:**
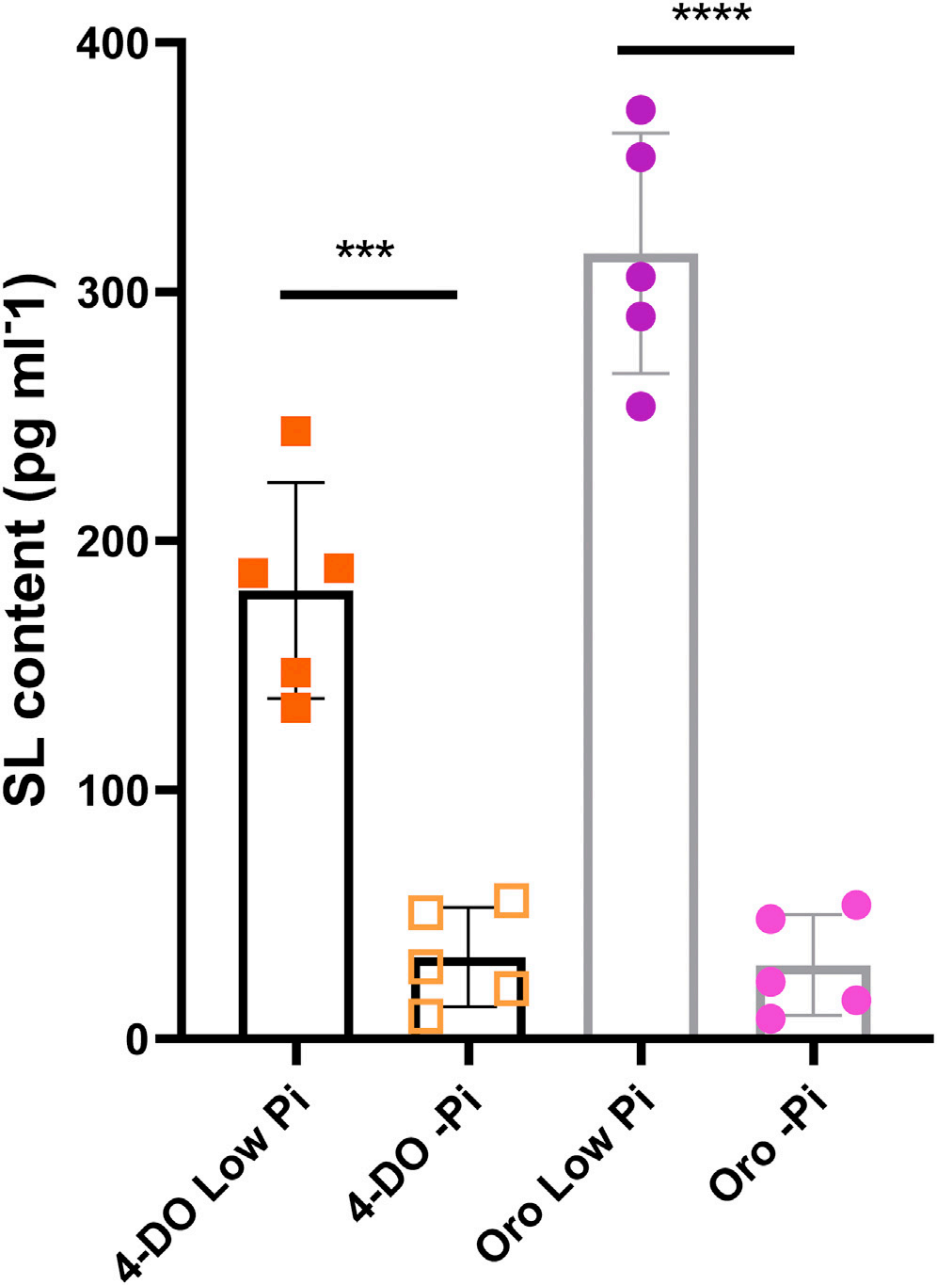
SL quantification of rice grown under Low Pi and -Pi conditions Data represent mean ± SD. *n*=5. Statistical analysis was performed using two-tail student *t*-test. Different letters denote significant differences (∗*p* < 0.05, ∗∗*p*< 0.01, ∗∗∗*p*< 0.001, ∗∗∗∗*p*< 0.0001). 4-DO, 4-deoxyorobanchol; Oro, Orobanchol.

***Note:*** Rice seedlings can be directly grown under low Pi conditions for two weeks, which generally showed higher SL content compared to -Pi conditions (Figure 2).

### Preconditioning of *Striga hermonthica* seeds

**Timing: ∼10 days**

The bioactivity of root-released SLs can be evaluated on the parasitic seeds of the *Orobanchaceae* family, as seed germination stimulants. However, to be responsive to external chemical signals, the parasitic seeds require pre-conditioning steps (Jamil et al., 2012) mimicking a humid and warm environment (see steps below).

10. Cleaning of *Striga s*eeds.
  a. Place 1 g *Striga* seeds in a 50 mL falcon tube with 40 mL tap water. Soak the seeds in water and agitate them on a rotator for 30 min.
  b. In a new 50 mL falcon tube, add 15 mL of 60% sucrose, followed by 10 mL of 40% sucrose solution added drop-by-drop.
    **CRITICAL:** Make sure to not disturb the sucrose gradient.
  c. Transfer gently 20 mL of the water-soaked-seeds to the sucrose-containing tube, without disturbing the gradient.
  d. Centrifuge at 1,356 × *g* for 10 min and thereafter collect the *Striga* seeds from the inner phase with the help of pipette in a vacuum assembly.
  e. Remove the sucrose from the seeds by 5–6 times successive washings with sterilized Milli-Q water. Let the wet seeds entirely dry in a laminar flow cabinet.
    ***Note:*** This drying step takes 12–42 h.
    **Pause point:** Keep the cleaned seeds in the Petri dish at room temperature (23°C–25°C) for long-term storage.

11. Pre-conditioning.
  a. Place the cleaned *Striga* seeds (∼5 mg/12 glass fiber filter discs) in a 50 mL tube.
  b. Add 20 mL 50% bleach solution (sterilized Milli-Q water+commercial bleach; 1:1 v/v) into the tube.
  c. Shake the tube in a rotator for 7 min.
    **CRITICAL:** Please do not keep more than 7 min because a long-term exposure can be lethal for the seeds.
  d. Transfer the seeds to a vacuum assembly to wash away the bleach by 5–6 successive washings with sterilized Milli-Q water in a laminar flow cabinet and dry the seeds entirely.
  e. Place twelve 9 mm sterilized glass fiber filter paper discs on a round glass Petri dish plate and spread evenly ∼50–100 *Striga* seeds on each disc.
  f. Put a sterilized 9 cm diameter Whatman filter paper in a 9 cm round plastic Petri dish plate and add 3 mL sterilized Milli-Q water on it.
  g. Transfer the above-mentioned (f) glass fiber filter discs with *Striga* seeds (12 discs per plate) and seal the plate with parafilm.
  h. Cover the plate(s) with aluminum foil and place the plate in an inverted position (keep the seeds upside down) in the incubator at 30°C for 10 days.
    **CRITICAL:** As water exposure will disturb *Striga* seed germination, please keep the plate inverted.

## Key resources table

**Table:** 

REAGENT or RESOURCE	SOURCE	IDENTIFIER
**Chemicals, peptides, and recombinant proteins**		

Methanol	VWR (≥99.9% (by GC), HiPerSolv CHROMANORM® for LC-MS)	Cat#83638
Water	VWR (HiPerSolv CHROMANORM® for LC-MS)	Cat#83645
Milli-Q Water	Merck (Quantum® Polishing Cartridge)	QTUM0TEX1
Ethyl Acetate	VWR (≥99.8%, HiPerSolv CHROMANORM® for HPLC)	Cat#83621
Acetonitrile	Fisher Scientific (Optima™ LC/MS Grade)	Cat#A955
Formic acid	Merck (98%–100% for LC-MS LiChropur®)	Cat#533002
4-deoxyorobanchol	OlChemIm	Cat#0257141
Orobanchol	OlChemIm	Cat#0256701
*rac*-GR24	Strigolab	https://strigolab.eu/
D_6_-5-deoxystrigol	The University of Tokyo	https://www.u-tokyo.ac.jp/focus/en/people/people001154.html
Ammonium nitrate	Fisher Scientific US	Cat#A676212
Magnesium sulfate heptahydrate	GOLD BIOTECH	Cat#M-020-1
Iron(II) sulfate heptahydrate, plant cell culture tested	Sigma-Aldrich	Cat#F8263-1KG
Ethylenediaminetetraacetic acid disodium salt dihydrate (EDTA)	Sigma-Aldrich	Cat#E6635-1KG
Calcium chloride dihyd 99% acs	Sigma-Aldrich	Cat#223506-500G
Potassium nitrate	Fisher Scientific US	Cat#MK6715212
Potassium phosphate dibasic trihydrate 2ReagentPlus(R), >=99.0%	Sigma-Aldrich	Cat#P5504-5KG
Boric Acid	Sigma-Aldrich	Cat#B6768
Manganese chloride tetrahydrate	ACROS ORGANICS	Cat#205895000
Copper Sulfate pentahydrate	Sigma-Aldrich	Cat#C8027
Zinc Chloride	Sigma-Aldrich	Cat#793523
Sodium molybdate	ACROS ORGANICS	Cat#206375000
Murashige & Skoog (MS) Basal Medium	Sigma-Aldrich	Cat#M5524-50L
Grade 4 qualitative filter papers, Whatman™, 90 mm, 1 cm thickness	A-VWR, Part of Avantor	Cat#512-1026
Plastic round Petri dish (PETRI DISH 90 × 16.2 MM)	VWR INTERNATIONAL, LTD-UK	Cat#391-0443
Silver sand	Hanson	https://www.hanson-packedproducts.co.uk/en/products/base-aggregates/silver-sand
Pierce™ FlexMix™ Calibration Solution	Thermo Scientific	Cat#A39239
50 mL black falcon tube	Heathrow Scientific	Cat#518520
1.5 mL Eppendorf tube	Eppendorf, North America	Cat#022363204
1.5 mL Black Eppendorf tube	Argos Technologies™	Cat#5087954
8 mL brown glass vial	VWR North America	Cat#548-0889
Cap of 8 mL glass vial	VWR North America	Cat#548-0862
1.5 mL glass autosampler vial	VWR North America	Cat#VWRI548-0030
Cap of 1.5 mL glass autosampler vial	VWR North America	Cat#89239-018
0.22 μm filter	Thermo Scientific	Cat#00215484
Commercial bleach (sodium hypochlorite)	Clorox® Bleach	https://www.cloroxarabia.com/en/products/clorox-bleach/original/
Tween-20	Thermo Scientific	Cat# 85113

**Experimental models: Organisms/strains**		

*Oryza sativa:* Nipponbare 3-week-old seedlings	KAUST	Butt et al. (2018)https://doi.org/10.1186/s12870-018-1387-1
*Oryza sativa: d17* (background Nipponbare) 3-week-old seedlings	KAUST	Butt et al. (2018)https://doi.org/10.1186/s12870-018-1387-1
*Pennisetum glaucum*: 29Aw 7-week-old plants	International Crops Research Institute for the Semi-Arid Tropics (ICRISAT)	Dayou et al. (2021)https://doi.org/10.1017/wsc.2021.12

**Software and algorithms**		

Xcalibur™ Software	Thermo Fisher Scientific	OPTON-30965
LAS-EZ-V3-0 software	Leica Microsystems	https://www.leica-microsystems.com/products/microscope-software/p/leica-las-ez/
SeedQuant	KAUST	https://braguyjm.github.io/SeedQuant2/
MultiQuant 2.1	SCIEX	https://sciex.com/products/software/multiquant-software

**Other**		

Hypersil GOLD™ C_18_ Selectivity HPLC Columns	Thermo Scientific	25003-032130
SPE C_18_-Fast (500 mg/3 mL)	SEClute™	5138758
Leica LED3000 R mounted with a CCD camera (Leica Microsystems)	Leica Microsystems	https://www.leica-microsystems.com/products/microscope-accessories/p/leica-led3000-bli/
UHPLC-Orbitrap ID-X Tribrid Mass Spectrometer	Thermo Fisher Scientific	https://www.thermofisher.com/sa/en/home/industrial/mass-spectrometry/liquid-chromatography-mass-spectrometry-lc-ms/lc-ms-systems/orbitrap-lc-ms/orbitrap-tribrid-mass-spectrometers/orbitrap-iq-x.html
HPLC-triple quadrupole/linear ion trap instrument (QTRAP5500)	AB Sciex	https://sciex.com/products/mass-spectrometers/qtrap-systems/qtrap-5500-system
UHPLC- Triple-Stage Quadrupole Mass Spectrometer (TSQ-Altis^TM^)	Thermo Fisher Scientific	https://www.thermofisher.com/order/catalog/product/TSQ02-10002

## Step-by-step method details

### SL collection and extraction

**Timing: ∼10 h**

Under phosphate starvation, roots of hydroponically grown plants continuously release SLs into the medium. Therefore, we recommend to let SLs accumulate in the medium for 6 h and then collect it (Wang et al., 2019): we usually refresh the solution in the early morning and collect the medium for SL extraction 6 h later.

***Optional:*** For plants grown in the sand system, we recommend to collect the exudate 24 h after pre-washing the pots with 1 L –Pi half-strength modified Hoagland solution (Jamil et al., 2014).

1. Root exudates collection.
  a. In the early morning, prepare fresh –Pi/Low Pi half-strength modified Hoagland solution with pH adjusted to ∼5.8.
  b. Refresh the medium (50 mL) of each tube containing rice seedlings and wait for 6 h.
  c. After 6 h, remove the plants from the tubes and store the collected medium with 50 mL tubes on ice.

***Alternatives:*** When using the sand system, we recommend to collect 500 –1,000 mL root exudates by adding –Pi/Low Pi half-strength modified Hoagland solution on the top of each pot and collect the flow-through, from the bottom of the pot, in 1 L plastic bottle.

***Note:*** During the collection, protect the collected root exudates from light to avoid SL degradation. We also recommend to extract the collected samples as soon as possible without any delay, as SLs degrade quickly in the water when exposed to light and high temperature.

***Optional:*** Transfer the seedlings to a new 50 mL tubes containing fresh half-strength modified Hoagland solutions for a second collection in case something goes wrong during the extraction.

***Optional:*** At this stage, plant tissues can be also collected for SL or transcript analysis. They should be immediately frozen in liquid nitrogen and stored at −80°C.

2. SL extraction from root exudates.
  a. Keep the collected root exudates on ice. In the lab, spike each sample, collected both from hydroponic and sand, with 0.672 ng of D_6_-5-deoxystrigol - used as internal standard (IS).
    ***Alternatives:*** The internal standard can be replaced by using the SL analog GR24 (1 ng).
    ***Note:*** If the collected exudate is to be applied to parasitic seeds, do not add internal standard. The latter will trigger high germination!
  b. Pre-condition Fast SPE C_18_ column (500 mg/3 mL; GracePure), connected with SPE Vacuum Manifold with 3 mL of methanol, followed by 3 mL of Milli-Q water.
    ***Note:*** After conditioning, keep the columns wet with water and do not let them dry before sample loading! The pre-conditioning step enables a condition to enable the analyte adsorption. Wetting the adsorbent with a suitable solvent ensures reproducibility.
  c. Attach a 50 mL syringe to the pre-conditioned C_18_ column and load the collected root exudate samples (before you begin step 9) onto them. After enrichment, wash the C_18_ column with 3 mL Milli-Q water to remove impurities.
    ***Optional:*** To get better MS resolution and sensitivity for SL identification, we suggest to pool at least 500 mL–1,000 mL root exudate.
    ***Note:*** If the volume of root exudate exceeds 200 mL, we strongly advice to add a pre-step, using glass fiber filter paper through a vacuum assembly, to remove impurities and plant debris.
  d. Elute SLs twice, into two separate 8 mL brown glass vials: use 2 mL acetone to collect the first fraction A, followed by 3 mL acetone for the fraction B.
    ***Note:*** For the parasitic seeds bioassay, elute the collected exudate with 3 mL acetone only, as the parasitic weeds are much more sensitive than LC-MS.
    **Pause point:** Seal the collected samples with caps in the brown glass vials and keep the acetone extracted samples in −20°C refrigerator for 2-week storage or at −80°C for long-term storage.
    **CRITICAL:** Try to avoid direct light exposure during the extraction process.

3. SL re-extraction for LC-MS.
  a. Fully dry the fraction B (from SL collection and extraction step 2d; containing mostly acetone) under vacuum.
  b. Add 1 mL ethyl acetate into each vial containing the dried fraction B, and gently vortex for 5 s.
  c. Dry the fraction A (from SL collection and extraction step 2d; containing a mix of acetone and water) under vacuum, which results in ∼300 μL partially dry (acetone-water).
    ***Optional:*** If space allows it, please vacuum-dry Fraction A and B together.
  d. Transfer 1 mL of re-suspended fraction B in ethyl acetate to fraction A, and gently vortex for 5 s. Two layers with ethyl acetate (up) and water (down) will appear.
  e. Centrifuge the samples at 1,356 × *g* for 2 min at room temperature.
  f. Carefully transfer 750 μL from the upper-layer (SLs enriched organic phase) to a new 1.5 mL Eppendorf tube.
    **CRITICAL:** Do not touch the lower layer to avoid water contamination.
  g. Fully dry the samples using a speed vacuum at room temperature.
    **Pause point:** Keep the extracts in −20°C refrigerator if you do not want to process them immediately.
  h. Dissolve the dried samples in 100 μL of acetonitrile:water (25:75, v:v) and filter them gently through a 0.22 μm filter into a glass autosampler vial.
  i. Close the vial with the cap before LC-MS/MS analysis.
  j. For better detection, gently tap the bottom of the vial to remove the gases in the samples.
    **Pause point:** Keep the filtered samples in −20°C refrigerator for storage.

### SL identification by LC-MS/MS

**Timing: 23 min per sample**

**Figure 3:**
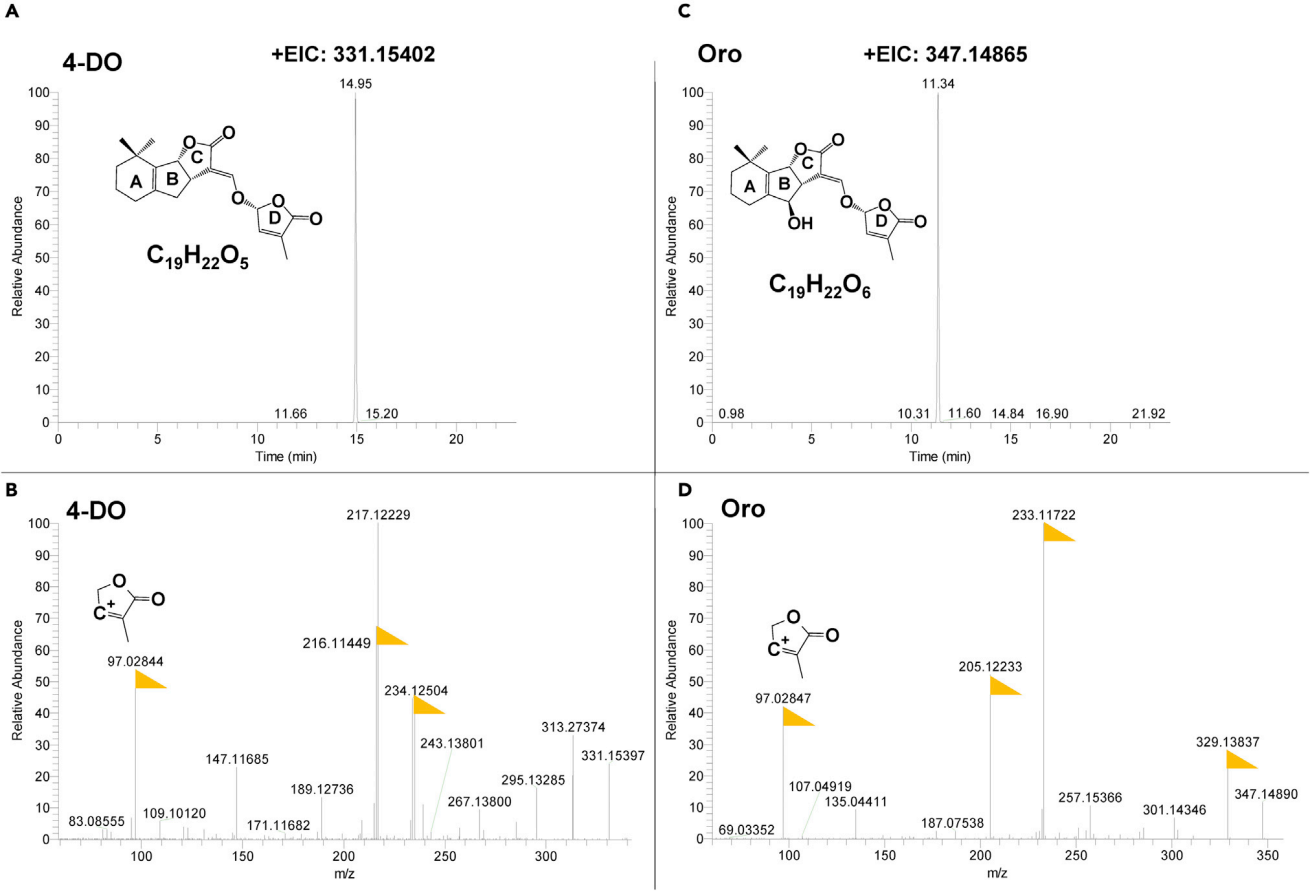
The chromatography of SL identification from authentic standards (A) Accurate mass of 4-deoxyorobanchol (4-DO). (B) MS/MS fragmentation of 4-DO (Seto et al., 2014). (C) Accurate mass of Orobanchol (Oro). (D) MS/MS fragmentation of Oro (Seto et al., 2014). The yellow flags point out the major fragments. +EIC = extracted ion chromatogram of positive mode.

**Figure 4:**
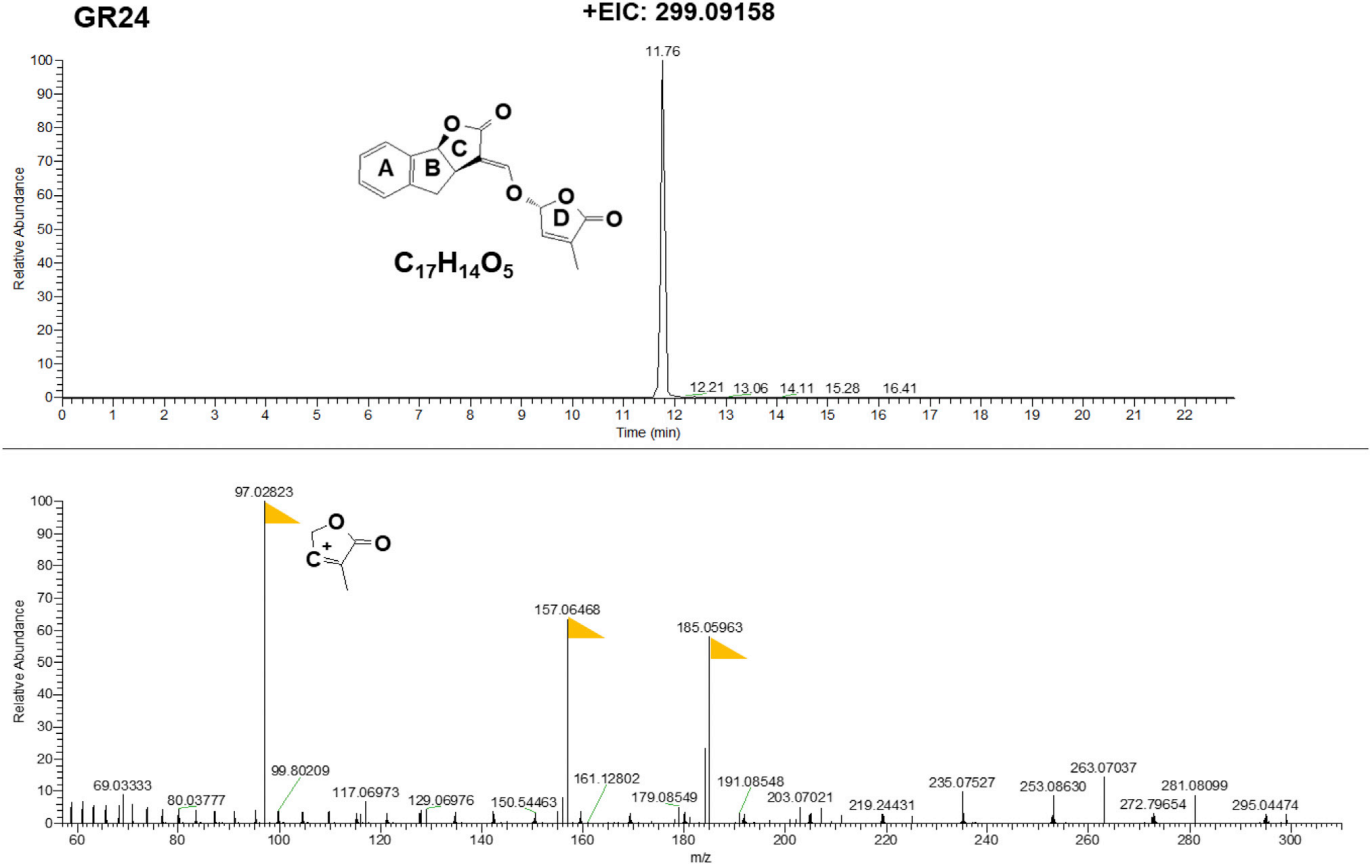
The chromatography of SL identification from authentic standard GR24 Accurate mass of GR24 (up). MS/MS fragmentation of GR24 (down) (Rebecca et al., 2020). The yellow flags point out the major fragments. +EIC = extracted ion chromatogram of positive mode.

Although SLs are divided into canonical and non-canonical SLs (Wang et al., 2021), they are generally traced and identified using the same methods. In our case, we use the IDX-Qrbitrap high-resolution Mass Spectrometry instrument, including the MS and MS/MS scans. The full scan MS analysis revealed the presence of the SL (4-deoxyorobanchol) at *m/z* 331.15402 (RT: 14.95 min). The MS/MS scan confirmed the presence of the common D-ring moiety fragment ion at *m/z* 97.02823, which corresponds to the conserved diagnostic product ion of SLs (Figures 3 and 4).

***Note:*** The IDX Orbitrap Mass Spectrometer is an analytical technique that contains three mass analyzers used for determining the *m*/*z* of small and big molecules. The Orbitrap IDX spectrometer could reach a high resolution (> 120,000) and reliable mass accuracy (<3 ppm mass error). The Mass spectrometer was calibrated using a purchasable “Calibration Mix ESI (Thermo Scientific)” and by following the manufacturer’s guidelines. Electrospray ionization in positive mode (ESI+) was applied for the studied compounds, using the following parameters: vaporized temperature = 100°C, voltage = 3,500 V, sheath gas = 30, auxiliary gas: 15, ion source fragmentation = 35 V, capillary temperature = 300°C. 10 μl of each sample were injected through a loop injection to a C_18_ column using an independent UPLC pump.

***Note:*** The separation of the studied metabolites was performed on a Hypersil GOLD™ C_18_ Selectivity HPLC Columns (150 × 4.6 mm; 3 μm) maintained at 35°C.

***Note:*** The mobile phases consisted of (A) 100% LC-MS grade water + 0.1% formic acid and (B) 100% Acetonitrile + 0.1% formic acid (Table 3). A gradient elution method was used (Table 4).

***Note:*** Identification of SLs is performed using an UHPLC system coupled with an Orbitrap ID-X Tribrid Mass Spectrometer, which runs in positive and negative modes. The MS parameters are listed in Table 5 and the parameters MS/MS are listed in Table 6.

***Note:*** Overall cycle time per sample is 23 min.

4. Prepare solvents as described in the Table 3.
5. Install the running solvent lines into the UHPLC solvent reservoirs.
6. Purge the solvent lines for 5 min.
7. Equilibrate the Orbitrap ID-X Tribrid system as shown in Tables 5 and 6.
8. Create a batch table.
9. Analyze samples in both positive and negative ion modes.

***Note:*** Injection volumes can vary from 5 to 10 μL, depending on sample concentrations.

**CRITICAL:** To prevent a shift in the retention time(s), the same batch of samples should be analyzed within 48 h in the same run.

10. Data processing by using Xcalibur™ Software.
11. Elemental composition to Calculate the theoretical accurate mass, e.g., 331.15400 for 4-DO, and search from the mass data using Xcalibur™ Software with 5 ppm mass tolerance (Figure 3).
12. Check MS/MS data to make sure to identify with the authentic standard (Figure 3).

**Figure 5:**
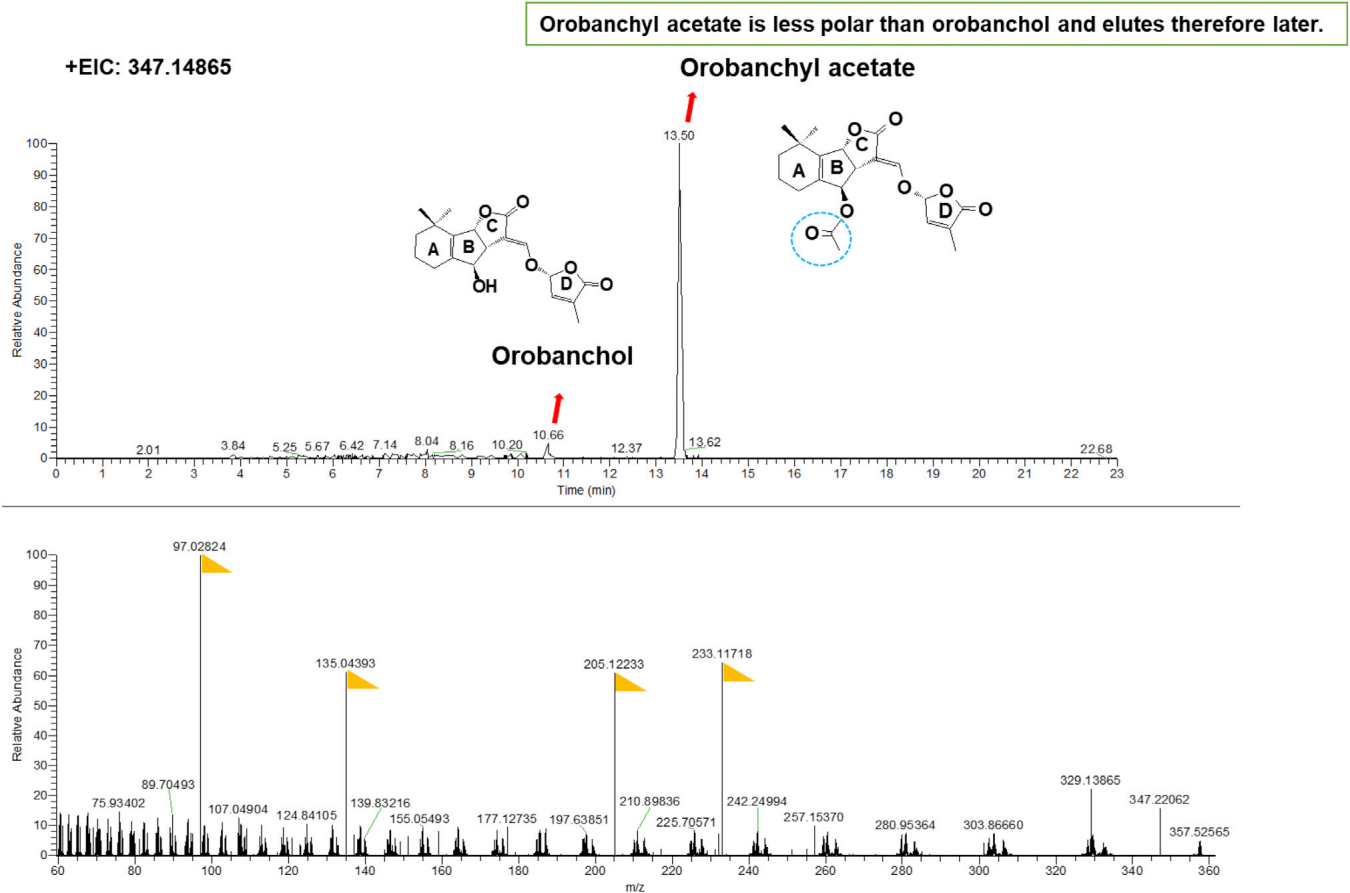
The chromatography of SL identification from pearl millet exudate Accurate mass of Orobanchol (up). MS/MS fragmentation of Orobanchyl acetate (down). The yellow flags point out the major fragments. +EIC= Extracted ion chromatogram of positive mode.

***Note:*** We provide the MS/MS spectrum of authentic 4-DO, Oro, and GR24 which can be used as references.

***Note:*** In Figure 5, the Oro and Orobanchyl acetate showed the same MS/MS, which could be distinguished by retention time.

**Table 3:** Solvent composition

Reagent	Final concentration	Amount
(A) Water	N/A	500 mL
(B) Acetonitrile	N/A	500 mL
Formic acid	0.1% (V/V)

**Table 4:** LC gradient condition for SL identification

Time	Flow (mL/min)	%A	%B
0	0.5	75	25
15	0.5	0	100
20	0.5	0	100
21	0.5	75	25
23	0.5	75	25

**Table 5:** General settings of MS

Parameters	Values
Ionization	Heated Electrospray Ionization
Spray Voltage	Static
Positive Ion (V)	3,500
Negative Ion (V)	2,500
Gas Mode	Static
Sheath Gas (Arb)	60
Aux Gas (Arb)	15
Sweep Gas (Arb)	2
Ion Transfer Tube Temperature (°C)	350
Vaporized Temperature (°C)	400
Detector Type	Orbitrap
Orbitrap Resolution	120000
Use Quadrupole Isolation	TRUE
Scan Range (m/z)	50–500
RF Lens (%)	60
AGC Target	Standard
Microscans	1
Data Type	Centroid

**Table 6:** General settings of MS/MS

Parameters	Values
Isolation Mode	Quadrupole
Isolation Window (m/z)	1.6
Isolation Offset	Off
Activation Type	HCD
Collision Energy Mode	Assisted
HCD Assisted Collision Energies (%)	10,20,30,40
Detection Type	Orbitrap
Orbitrap Resolution	60,000
Scan Range Mode	Auto
AGC Target	Standard
Microscans	1
Data Type	Centroid

### SL quantification by LC-MS/MS

**Timing: 25 min per sample**

**Figure 6:**
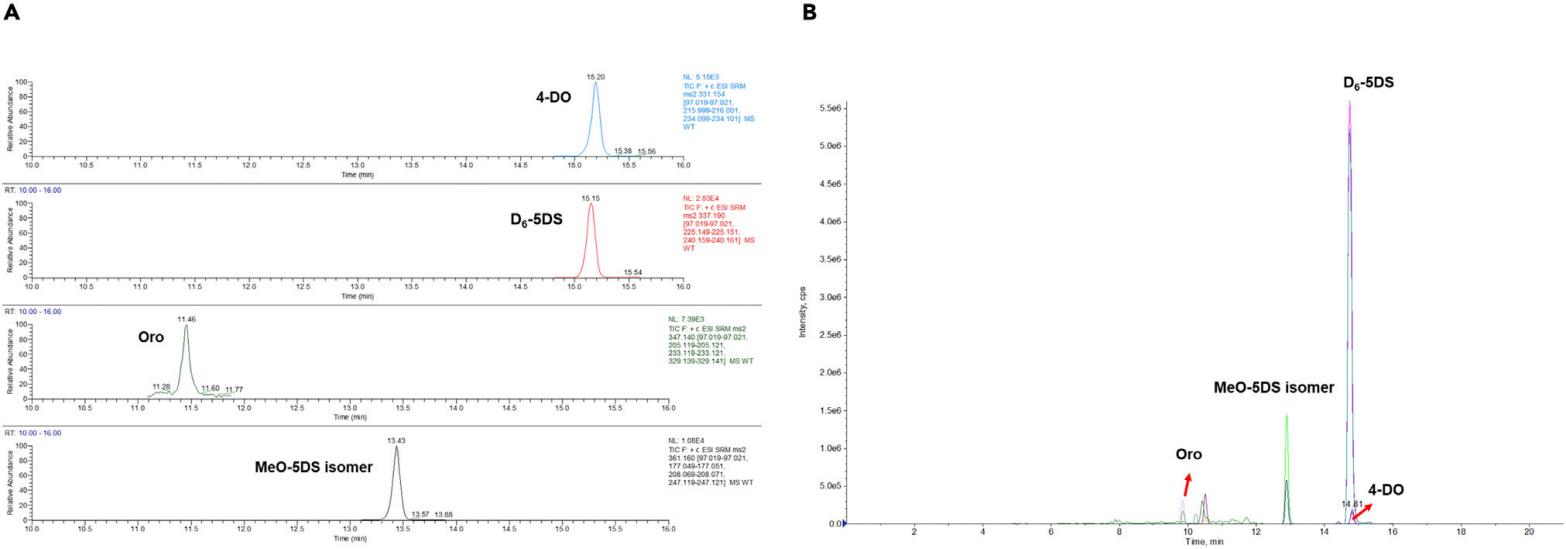
The representative UHPLC–MS/MS chromatograms. Overview of MRM chromatography from (A) TSQ-Altis and (B) QTRAP5500 Mass spectrometer, respectively. 4-DO, 4-deoxyorobanchol; Oro, Orobanchol; MeO-5DS, methoxy-5-deoxystrigol; D_6_-5DS, isotopically labeled (D_6_)-5-deoxystrigol.

A highly specific and sensitive method has been accomplished to selectively quantify compounds within complex mixtures by quadrupole MS analyzer. Quantification of SLs was performed by the UHPLC-TSQ-Altis Mass spectrometer using Multiple Reaction Monitoring (MRM) experiment (Figure 6A).

***Note:*** The TSQ Altis is a triple stage quadrupole Mass Spectrometer that determines the *m*/*z* of small molecules and which provides the best transmission and peak shape. This instrument has an electrospray ionization source and is capable of scan functions, such as Selective Ion Monitoring (SIM), Selective/Multiple Reaction Monitoring (S/MRM), and precursor ion scanning. The Mass spectrometer was calibrated using a purchasable “Calibration Mix ESI (Thermo Scientific)” by following the manufacturer’s guidelines.

***Note:*** The separation of metabolites is on a Hypersil GOLD™ C_18_ Selectivity HPLC Columns (150 × 4.6 mm; 3 μm) maintained at 35°C.

***Note:*** The mobile phases consist of (A) 100% LC-MS grade water + 0.1% formic acid and (B) 100% Acetonitrile plus 0.1% formic acid are used for gradient elution (Tables 7 and 8).

***Note:*** Identification of SLs is performed using an UHPLC system coupled with a Triple-Stage Quadrupole Mass Spectrometer (TSQ-Altis^TM^), which runs in positive mode. The general MS parameters of MRM are listed in Table 9, and the ion transitions of SLs are listed in Table 10.

***Note:*** Overall cycle time per sample is 25 min.

***Optional:*** The MRM parameters of HPLC-triple quadrupole/linear ion trap instrument (QTRAP5500) are listed in Table 11 that shown in Figure 6B while the settings of ion transitions are the same as in Table 10.

13. Prepare solvents as described in the Table 7.
14. Install the running solvent lines into the UHPLC solvent reservoirs.
15. Purge the solvent lines for 5 min.
16. Equilibrate the Triple-Stage Quadrupole Mass Spectrometer (TSQ-Altis^TM^) system as shown in Table 9.
17. Create a batch table and insert pooled QC samples once every ten times.

***Optional:*** Insert authentic standard samples, if having, in the beginning and end of running.

18. Analyze samples in positive ion modes.

***Note:*** Injection volumes can be changed from 5 to 10 μL, depending on the sample concentrations.

**CRITICAL:** To prevent retention time-shifting, the same batch of samples should be analyzed within 48 h in the same run.

19. Analyze data in Xcalibur™ Software.
20. Create a processing method based on the MRM results before batch analysis.
21. Output automatic integrative peak area results as a CSV file for calculation (see quantification and statistical analysis section).

**Table 7:** Solvent composition

Reagent	Final concentration	Amount
(A) Water	N/A	500 mL
(B) Acetonitrile	N/A	500 mL
Formic acid	0.1% (V/V)

**Table 8:** LC gradient condition for MRM analysis

Time	Flow (mL/min)	%A	%B
0	0.5	75	25
15	0.5	0	100
20	0.5	0	100
21	0.5	75	25
25	0.5	75	25

**Table 9:** MS parameter settings of SRM analysis

Parameters	Values
Ionization	Heated Electrospray Ionization
Spray Voltage	Static
Positive Ion (V)	4,000
Negative Ion (V)	3,500
Sheath Gas (Arb)	40
Aux Gas (Arb)	15
Sweep Gas (Arb)	5
Ion Transfer Tube Temperature (°C)	350
Vaporized Temperature (°C)	350
Cycle Time (Sec)	0.8
Q1 Resolution (FWHM)	0.2
Q3 Resolution (FWHM)	0.2
CID Gas (mTorr)	2
Chromatographic Peak Width (Sec)	6

**Table 10:** The MRM detection parameters of SLs

Strigolactones	Monitoring transitions (m/z; [M+H] ^+^)		
	Precursor ion	Diagnostic product ion	Confirming product ion
D_6_-5DS	337.19	97.02	240.16 **|** 222.15
GR24	299.08	97.02	157.06 **|** 185.05
4DO	331.15	97.02	234.1 **|** 216.0
Oro	347.14	97.02	329.14 **|** 233.12 **|** 205.12
MeO-5DS isomer	361.16	97.02	247.12 **|** 208.07 **|** 177.05
Oro-Acetate∗	389.15	97.02	329.14 **|** 233.12 **|** 205.12
	411.1∗ ∗[M+Na] ^+^	97.02	351.1 **|** 254.1

**Table 11:** The MRM parameters of HPLC-triple quadrupole/linear ion trap instrument (QTRAP5500)

Parameters	Values
Ionization	Turbo Spray
Spray Voltage	5,500
Curtain Gas (CUR)	20
Collision Gas (CAD)	Medium
Temperature (TEM)	400
Ion Source Gas 1 (GS1)	80
Ion Source Gas 2 (GS2)	70
Declustering Potential (DP)	60
Entrance Potential (EP)	12
Collision Energy (CE)	16
Collision Cell Exit Potential (CXP)	15

### SL bioactivity on *Striga* seed germination

**Timing: ∼30 h**

22. Preparation of collected SLs for bioassays.
  a. Take 400 μL of each SL-containing sample eluted by acetone (from SL collection and extraction step 2d) into 2 mL Eppendorf tube and add 400 μL sterilized Milli-Q water (1:1 dilution).
  b. Remove the acetone by speed vacuum for 60 min at room temperature (23°C–25°C) or preferably lower temperature.

**CRITICAL:** As acetone can affect the germination, make sure to keep enough time for evaporation. One can make a mark on the tube corresponding to 400 μL volume, to ensure the total evaporation of acetone in the sample.

***Optional:*** For the SL-containing samples collected from 500 mL to 1,000 mL, try to dilute the sample into a 1:3 to 1:9 ratio for the bioassays.

**Figure 7:**
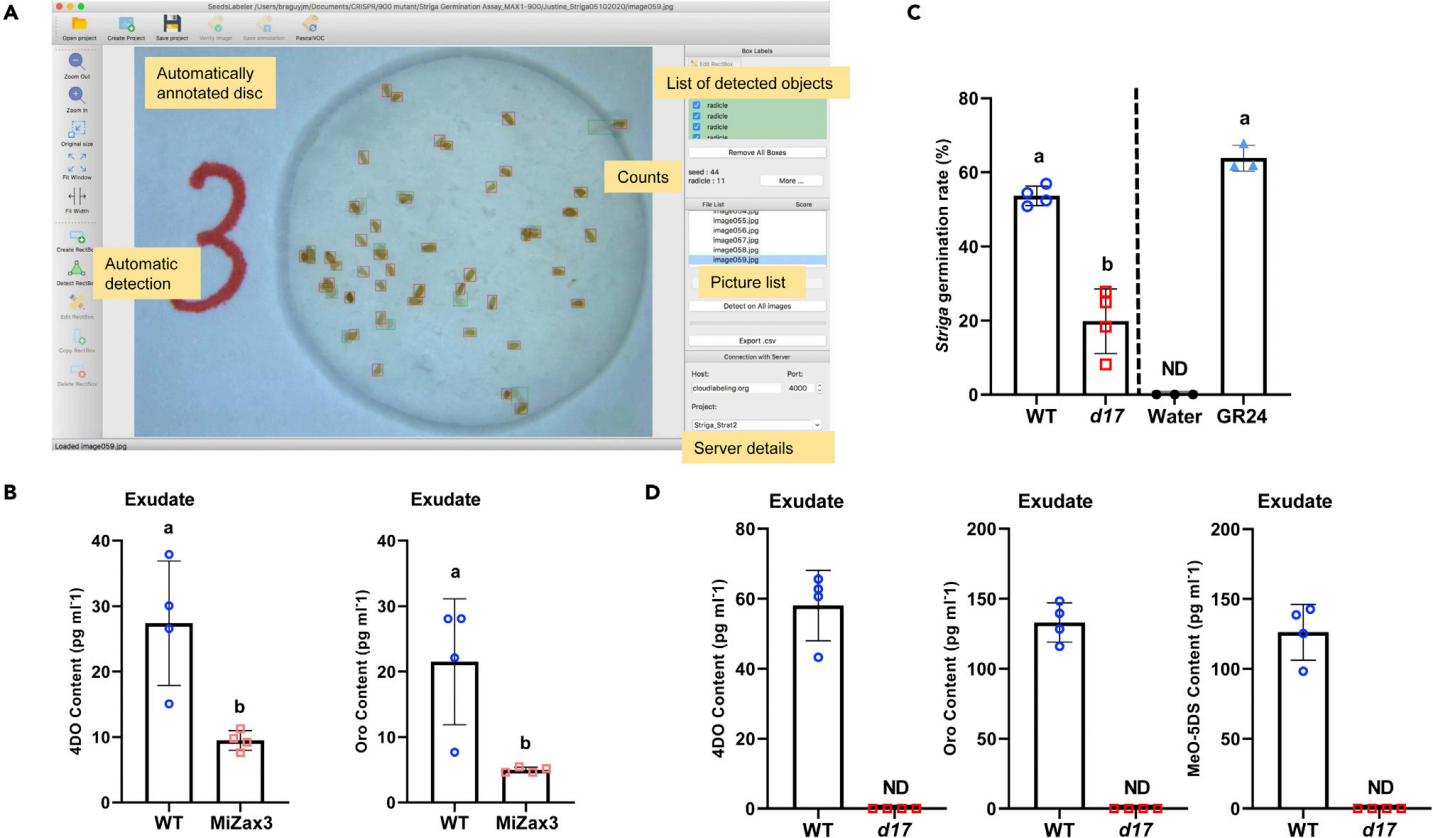
Expected outcome obtained from this protocol (A) Overview and annotation of SeedQuant software interface. (B) Evaluation of MiZax effect by SL quantification. (C) *Striga* bioassay results processed by SeedQuant. (D) SL quantification between two rice genotypes. Data represent mean ± SD. *n*=4. Statistical analysis was performed using One-way analysis of variance (ANOVA) and Tukey’s post hoc test. MiZax3, zaxinone mimics 3; WT, wild type; *d17*, *dwarf17,* a SL biosynthesis mutant. ND, not-detected
  d. Calculate the germination percentage by dividing germinated seeds over total seeds to evaluate the bioactivity of the plant released SLs.

23. *Striga* germination bioassays.
  a. Dry the 10-day-preconditioned *Striga* discs (from the section of preconditioning of Striga hermonthica seeds) in a laminar flow cabinet.
  b. Place a filter paper ring (∼1 cm wide) cut from a 9 cm diameter Whatman filter paper in the 9 cm plastic Petri dish plate.
  c. Transfer six *Striga* discs to each Petri dish plate in the middle of the ring.
    **CRITICAL:** Avoid touching the filter paper ring, as water will interfere with the seed germination.
  d. Apply 50 μL of aforementioned SL-containing sample (1:1 dilution) on each *Striga* disc and humidify the filter paper ring with 900 μL sterilized Milli-Q water.
  e. Seal the plates with parafilm and cover with aluminum foil. Incubate at 30°C for 24 h to germinate.
    **CRITICAL:** Please include a positive control (50 μL of 1 μM GR24) and negative control (50 μL of sterilized H_2_O) to ensure the quality of the experiment.

24. Scanning and counting.
  a. Take the incubated plates out of the incubator and open them to dry for 15 min.
    ***Note:*** If the discs are not dry, there will be reflected when the discs will be imaged.
  b. Capture the images of seed-containing discs individually with a Leica LED3000 R adjusted to 50% medium light, mounted with a CCD camera (Leica Microsystems) and store the images in a laptop.
    ***Note:*** The images were saved in 8-bit, 2,592 × 1,944 pixels and exported in JPEG format using the LAS-EZ-V3-0 software for image acquisition.
  c. Detect and count the germinated and non-germinated seeds with SeedQuant (Braguy et al., 2021) (Figure 7A).

## Expected outcomes

By using this protocol, we can unarguably identify endogenous SLs by comparing MS/MS pattern with authentic standards. In addition, we can evaluate the effect of a synthetic compound, such as the zaxinone and zaxinone mimics (MiZax), which regulate SL biosynthesis and release (Wang et al., 2019, 2020) (Figure 7B), as well as quantify the SL levels among different genotypes (Figure 7D). Finally, by applying collected root exudates to pre-conditioned *Striga* seeds – which is a much more sensitive method than LC-MS detection -, we can simply correlate bioassays (Figure 7C) with LC-MS results, and confirm the released SL’s bioactivity in the rhizosphere.

## Quantification and statistical analysis

For the quantification of analytes (Table 12), we calculate endogenous amounts by dividing the analyte peak area with the one of the internal standard (IS), which is represented as *Content (pg/mL) = (Analyte Peak Area/IS Peak Area)∗added IS (ng)∗1000/Sample collected Volume (mL)*.

**Table 12:** MRM raw data of LCMS used in the SL quantification between two rice genotypes

Sample	Analyte name	Analyte peak area (counts)	Analyte retention time (min)	IS peak name	IS peak area (counts)	IS retention time (min)	Content (pg/replicate)
*d17-1*	4-DeoxyOrobanchol	N/A	N/A	D_6_-5DS	5.97E+06	14.86	N/A
*d17-2*	4-DeoxyOrobanchol	N/A	N/A	D_6_-5DS	5.88E+06	14.83	N/A
*d17-3*	4-DeoxyOrobanchol	N/A	N/A	D_6-_5DS	4.85E+06	14.84	N/A
*d17-4*	4-DeoxyOrobanchol	N/A	N/A	D_6_-5DS	7.22E+06	14.84	N/A
WT-1	4-DeoxyOrobanchol	4.74E+05	14.9	D_6_-5DS	7.36E+06	14.86	43.28
WT-2	4-DeoxyOrobanchol	6.72E+05	14.9	D_6_-5DS	7.45E+06	14.86	60.62
WT-3	4-DeoxyOrobanchol	6.96E+05	14.93	D_6_-5DS	7.13E+06	14.88	65.62
WT-4	4-DeoxyOrobanchol	6.70E+05	14.91	D_6_-5DS	7.17E+06	14.86	62.79
*d17-1*	Orobanchol	N/A	N/A	D_6_-5DS	5.97E+06	14.86	N/A
*d17-2*	Orobanchol	N/A	N/A	D_6_-5DS	5.88E+06	14.83	N/A
*d17-3*	Orobanchol	N/A	N/A	D_6-_5DS	4.85E+06	14.84	N/A
*d17-4*	Orobanchol	N/A	N/A	D_6_-5DS	7.22E+06	14.84	N/A
WT-1	Orobanchol	1.41E+06	10.62	D_6_-5DS	7.36E+06	14.86	128.30
WT-2	Orobanchol	1.29E+06	10.61	D_6_-5DS	7.45E+06	14.86	115.97
WT-3	Orobanchol	1.57E+06	10.64	D_6_-5DS	7.13E+06	14.88	148.31
WT-4	Orobanchol	1.49E+06	10.61	D_6_-5DS	7.17E+06	14.86	139.61
*d17-1*	MeO-5DS	N/A	N/A	D_6_-5DS	5.97E+06	14.86	N/A
*d17-2*	MeO-5DS	N/A	N/A	D_6_-5DS	5.88E+06	14.83	N/A
*d17-3*	MeO-5DS	N/A	N/A	D_6-_5DS	4.85E+06	14.84	N/A
*d17-4*	MeO-5DS	N/A	N/A	D_6_-5DS	7.22E+06	14.84	N/A
WT-1	MeO-5DS	1.08E+06	13.01	D_6_-5DS	7.36E+06	14.86	98.35
WT-2	MeO-5DS	1.39E+06	13.01	D_6_-5DS	7.45E+06	14.86	125.18
WT-3	MeO-5DS	1.51E+06	13.03	D_6_-5DS	7.13E+06	14.88	142.65
WT-4	MeO-5DS	1.48E+06	13.01	D_6_-5DS	7.17E+06	14.86	138.67

Alternatively, if no IS is used, simply measure the peak area of the selected analyte and compare it to the total average peak area across all the studied genotypes.

Table 13 summarizes the raw data of Figure 7C, counted by SeedQuant. To determine the *Striga* germination percentage, we divide the number of radicals (corresponding to the number of germinated *Striga* seeds) by the total number of seeds (including germinated and non-germinated *Striga* seeds). Usually, we apply each collected sample to four *Striga* discs – four technical replicates - and count their average; thereafter, the *Striga* germination rate for each plant of the same genotype is averaged to give the final germination rate (Figure 7C).

**Table 13:** Example of SeedQuant raw data

Sample	Replicate	Radicle	Seed	Percentage of germinated (radical/seed)	Average
GR24	1	38	56	67.85714286	63.82134414
	2	29	47	61.70212766	
	3	39	63	61.9047619	
H_2_O	1	0	74	0	0
	2	0	74	0	
	3	0	72	0	
	4	0	53	0	
WT1	1	36	83	43.37349398	52.41200095
	2	31	51	60.78431373	
	3	40	68	58.82352941	
	4	28	60	46.66666667	
WT2	1	44	79	55.69620253	50.83424193
	2	37	79	46.83544304	
	3	29	56	51.78571429	
	4	25	51	49.01960784	
WT3	1	36	65	55.38461538	54.44710337
	2	36	69	52.17391304	
	3	32	60	53.33333333	
	4	33	58	56.89655172	
WT4	1	52	93	55.91397849	56.94924783
	2	23	48	47.91666667	
	3	43	65	66.15384615	
	4	37	64	57.8125	
*d17-1*	1	21	89	23.59550562	24.93116051
	2	32	84	38.0952381	
	3	13	59	22.03389831	
	4	12	75	16	
*d17-2*	1	3	82	3.658536585	8.170308197
	2	3	53	5.660377358	
	3	6	69	8.695652174	
	4	11	75	14.66666667	
*d17-3*	1	13	57	22.80701754	18.33589502
	2	16	75	21.33333333	
	3	12	74	16.21621622	
	4	10	77	12.98701299	
*d17-4*	1	17	53	32.0754717	27.78734863
	2	12	59	20.33898305	
	3	11	44	25	
	4	28	83	33.73493976	

## Limitations

Although this protocol can be applied to several plant species, the non-canonical SLs in the exudate are barely detected by LC-MS in general, due to the instability and lack of authentic standards. Undoubtedly, this could be partially solved by *Striga* bioassay on the basis of SL bioactivity. However, we cannot exclude the possibility of the presence of non-SL compounds that trigger parasitic plant seed germination, as observed in the rice *d17* mutant. These germinating stimulants should be referred to as SL-independent metabolites. Another limitation is coming from SeedQuant, which the counting accuracy of *Striga* germination counted is around 93%, which is comparable to the error rate of a trained scientist.

## Troubleshooting

### Problem 1

SL cannot be isolated for MS/MS (step 9 in SL identification by LC-MS/MS).

### Potential solution

We suggest adding a data-dependent MS/MS scan of the targeted SL ion after a full scan MS and to lower the threshold for the ion count to optimize the MS/MS fragmentation.

### Problem 2

SL content is lower than LC-MS detection (step 18 in SL quantification by LC-MS/MS).

### Potential solution

Make sure the plants are growing under phosphate deficient conditions. Else, you can pool two to three 50 mL sample tubes or increase the sample volume as one single biological replicate to enhance the SL concentration.

### Problem 3

*Striga* seeds did not germinate (step 23 in SL bioactivity on Striga seed germination).

### Potential solution

The lack of seed germination can have different reasons. 1) An inefficient pre-conditioning that can be verified by using the positive control GR24 (1 μM), which usually leads to >50% germination. 2) The presence of residual amount of acetone in the samples, which is toxic for the seeds.

### Problem 4

SeedQuant software does not respond (step 24 in SL bioactivity on Striga seed germination).

### Potential solution

Reopen the software and make sure the internet connection is active. If it still does not respond, it could be that the server itself is down, please do not hesitate to contact right away Justine.braguy@kaust.edu.sa or Silvio.giancola@kaust.edu.sa and share your issue.

### Problem 5

SeedQuant does not detect properly the germinated and non-germinated seeds (step 24 in SL bioactivity on Striga seed germination).

### Potential solution

SeedQuant seed detection can be impaired by the presence of plant debris or a different light intensity during the discs capture. The detection algorithm has reached its limitations, but a fine-tuning for your experimental conditions is possible. Contact Justine.braguy@kaust.edu.sa.

## Resource availability

### Lead contact

Further information and requests for resources and reagents should be directed to and will be fulfilled by the lead contact, Salim Al-Babili (salim.babili@kaust.edu.sa).

### Materials availability

All materials generated in this study are available from the lead contact upon completing a Materials Transfer Agreement.

## Data Availability

No unique datasets or codes were generated in this study.
